# Non-rotation of the Intestine: A Rare and Unexpected Cause of Bowel Obstruction in an Octogenarian

**DOI:** 10.7759/cureus.83971

**Published:** 2025-05-12

**Authors:** Andrew Kaler, Adam Khader, Anis Munazza, Jeannie F Rivers, Thomas A Miller

**Affiliations:** 1 Surgery, Virginia Commonwealth University School of Medicine, Richmond, USA; 2 Surgical Oncology, Central Virginia Veterans Affairs (VA) Health Care System, Richmond, USA; 3 Surgical Oncology, Virginia Commonwealth University Health System, Richmond, USA; 4 Radiology, Central Virginia Veterans Affairs (VA) Health Care System, Richmond, USA; 5 Radiology, Virginia Commonwealth University Health System, Richmond, USA; 6 Surgery, Central Virginia Veterans Affairs (VA) Health Care System, Richmond, USA; 7 Surgery, Virginia Commonwealth University Health System, Richmond, USA

**Keywords:** bowel obstruction, ladd’s bands, ladd’s procedure, malrotation, non-rotation, volvulus

## Abstract

An 85-year-old male presented to our emergency department with a three-day history of nausea, vomiting, diffuse lower abdominal pain, and diarrhea. His last bowel movement was in the morning prior to the evaluation. Physical exam demonstrated diffuse abdominal tenderness without peritoneal signs. CT scanning of the abdomen and pelvis (without contrast) was concerning for low-grade obstruction. He was observed for the next 24 hours. Although some improvement occurred, an elevated serum lactate and deteriorating renal function resulted in the decision to perform diagnostic laparoscopy to assess the presumed small bowel obstruction. Operative findings suggested the possibility of malrotation, so an open laparotomy was performed. This operation demonstrated the presence of Ladd’s bands with torsion of the small bowel. The Ladd’s bands were lysed, the detorsed small bowel was then placed on the right side of the abdomen, and the colon was placed on the left side with the cecum in the left upper quadrant, allowing broadening of the mesentery. The appendix was also removed in accordance with Ladd’s procedure. The patient tolerated the operation well but required extended postoperative care to manage his renal failure and new-onset atrial fibrillation. The patient was discharged on the 10th postoperative day and followed for the next year and a half without subsequent problems.

## Introduction

Intestinal malrotation is a relatively uncommon clinical condition. It has been estimated to occur with a frequency of one in 500 live births, but only one in every 6000 patients with malrotation will have symptoms [1,2]. Most patients (75-85%) with this condition who manifest clinically will do so within the first year of life, with nearly 40% in the first week of life [3]. The typical clinical presentation is midgut volvulus, which occurs secondary to the narrow base of the mesentery resulting from the malrotation prone to clockwise twisting [3].

While clinically important malrotation is typically a pediatric disease that usually occurs in the first year of life, it can also present throughout childhood, so it must not be forgotten that it can express itself for the first time at any age [4]. We present a patient who first demonstrated evidence of congenital malrotation at 85 years of age. His particular situation indicates that malrotation can remain clinically dormant for many, many decades. Thus, while rare later in life, the presence of this anatomic abnormality must not be forgotten and can be responsible for something as common as bowel obstruction, which turned out to be the circumstance in our patient. To our knowledge, this patient is the oldest individual reported to have a malrotation problem.

## Case presentation

**Figure 1:**
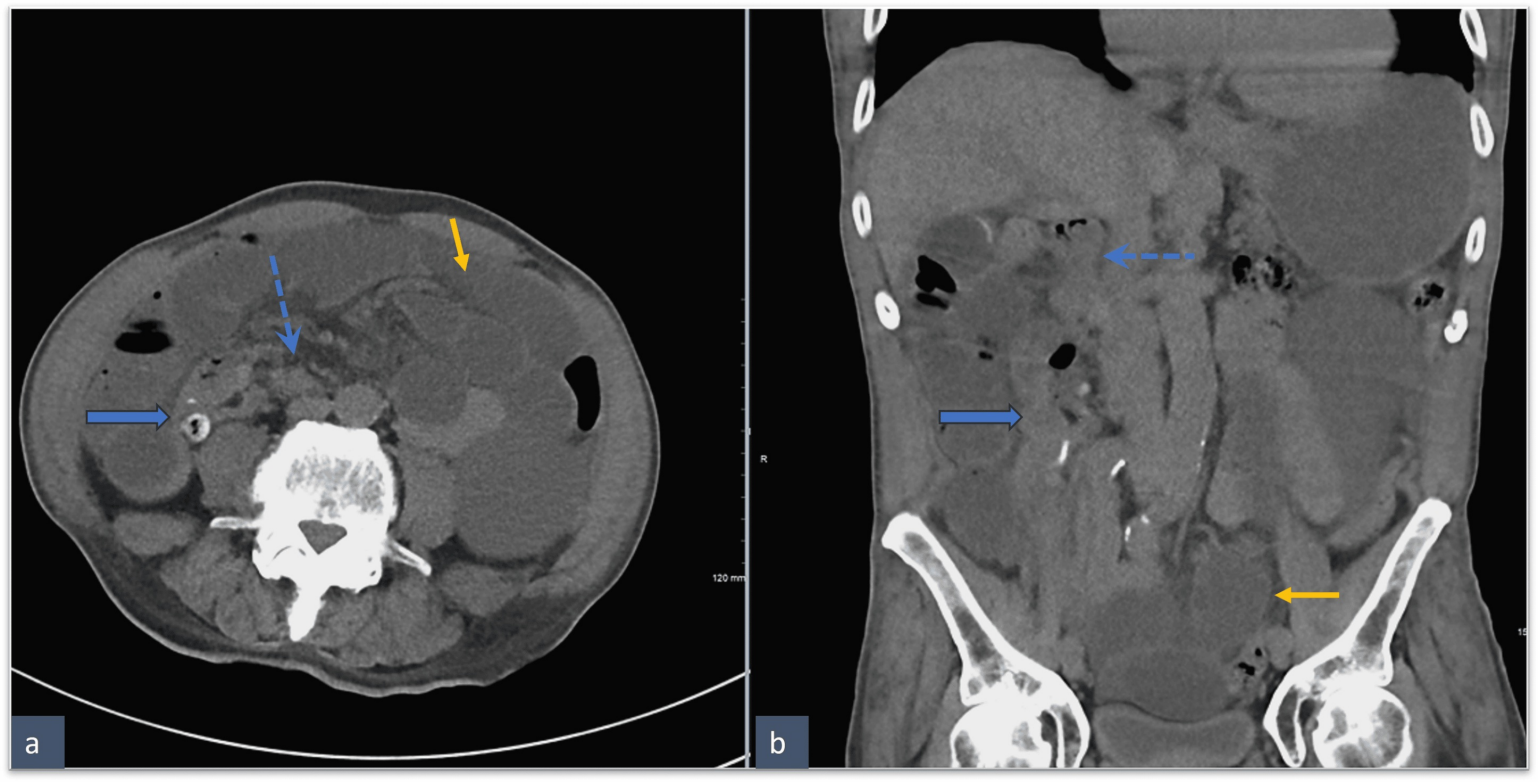
Non-contrast CT of the abdomen and pelvis at presentation Dilated small bowel loops are compatible with high-grade obstruction marked by the solid yellow arrow. The blue dashed arrow marks the duodenum. The blue block arrow marks the cecum with a diverticulum. CT: computed tomography

**Figure 2:**
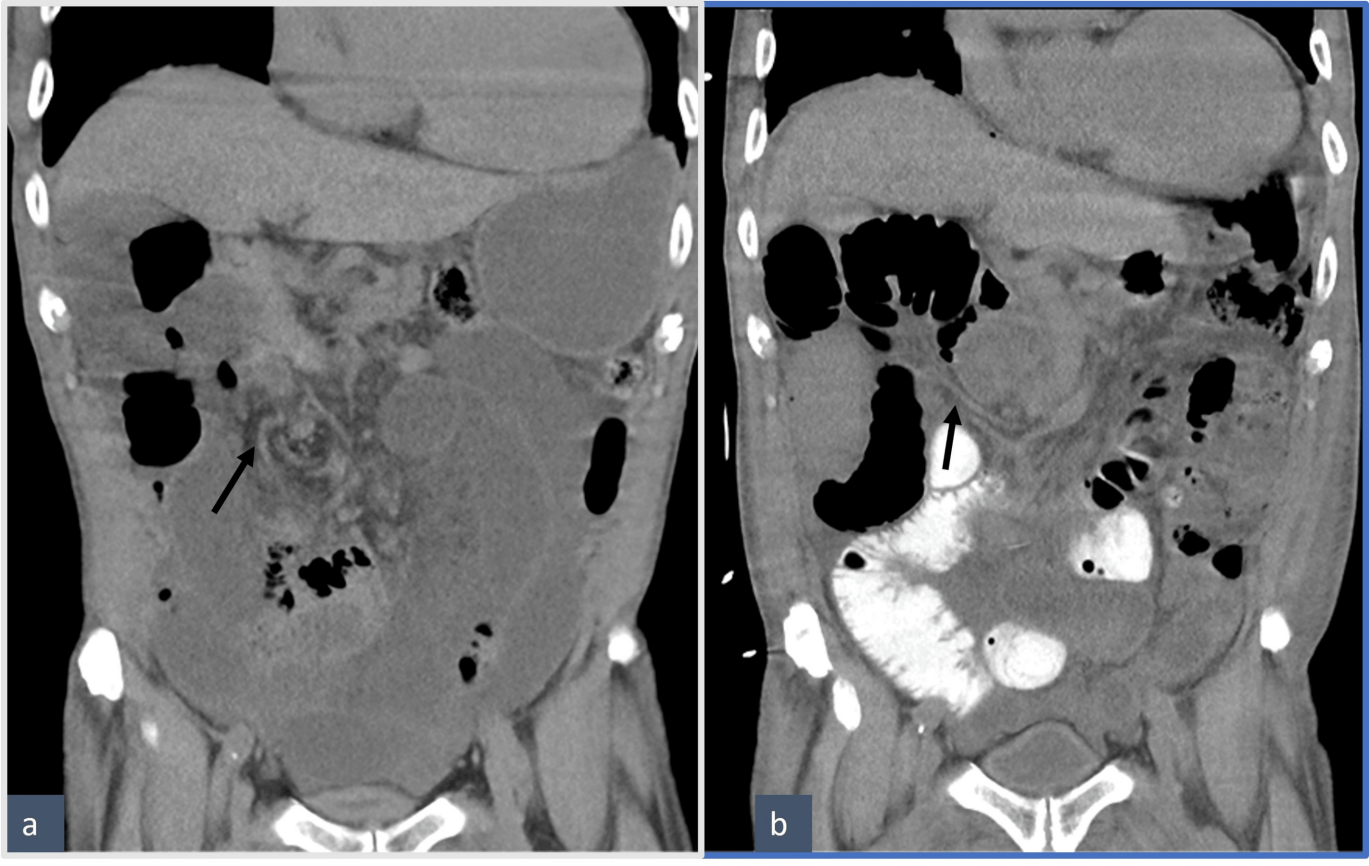
Pre- (a) and post-surgery (b) coronal non-IV contrast images The post-surgery image is with oral contrast (b). Arrows indicate the "whirl" appearance of the mesenteric vasculature pre-surgery (a) as opposed to the more linear appearance after surgery (b). IV: intravenous

**Figure 3:**
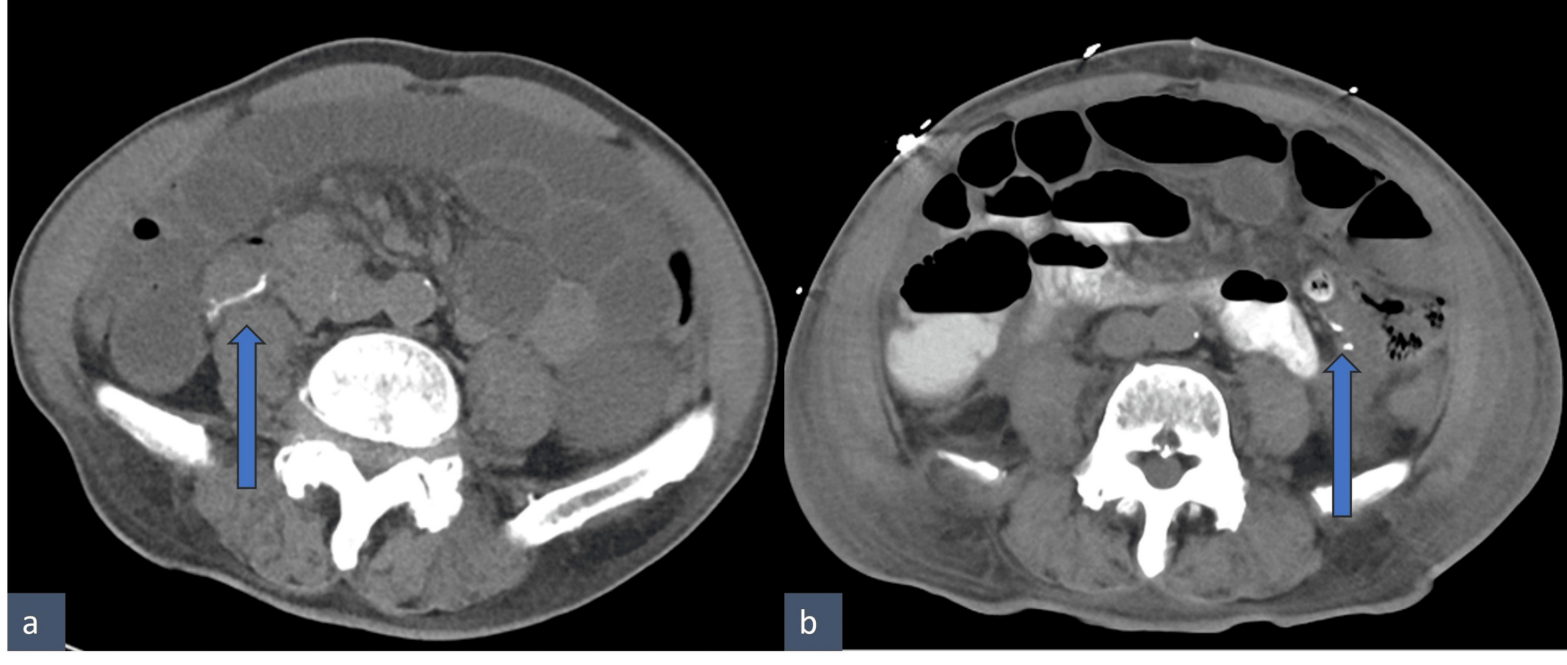
Axial pre- (a) and post-surgery (b) CT abdomen Cecum, marked by blue arrows, is in the medial location, inferior to the duodenum pre-surgery and lateral on the left of the abdomen post-surgery. CT: computed tomography

An 85-year-old male presented to the emergency department of our Veterans Affairs Hospital with three days of nausea, vomiting, diffuse lower abdominal pain, and diarrhea. He also reported that he had not been able to eat or drink secondary to his symptoms and had experienced a decrease in urine output. His last bowel movement was the morning prior to his arrival in the emergency room, and he endorsed passing flatus the night before. The patient reported no prior history of abdominal surgery and had no post-surgical changes on his abdomen that would suggest otherwise. The only pertinent history relative to presenting symptoms was a 1 cm cecal polyp with low-grade dysplasia noted on colonoscopy five years previously. The physical exam revealed diffuse abdominal tenderness with no peritoneal signs. Laboratory findings were significant for an elevated lactate of 3.3 (normal 0-2) and an acute on chronic elevation of renal function tests (creatinine 2.8 (normal <1.35 mg/dL); BUN 32 (normal <24 mg/dL)). CT scanning of the abdomen and pelvis without contrast was performed, and the radiologist interpreted the results as mild diffuse dilatation of the small bowel concerning for a low-grade obstruction (Figure 1, Figure 2a, and Figure 3a). The initial read by the radiologist described an anastomotic stricture from a presumed right hemicolectomy, based on the bowel orientation (Figure 1, Figure 2a, and Figure 3a). However, this presumed hemicolectomy was inconsistent with the patient’s history. A nasogastric tube was placed, and 750 mL of bilious stomach content was drained, immediately relieving the patient.

**Figure 4:**
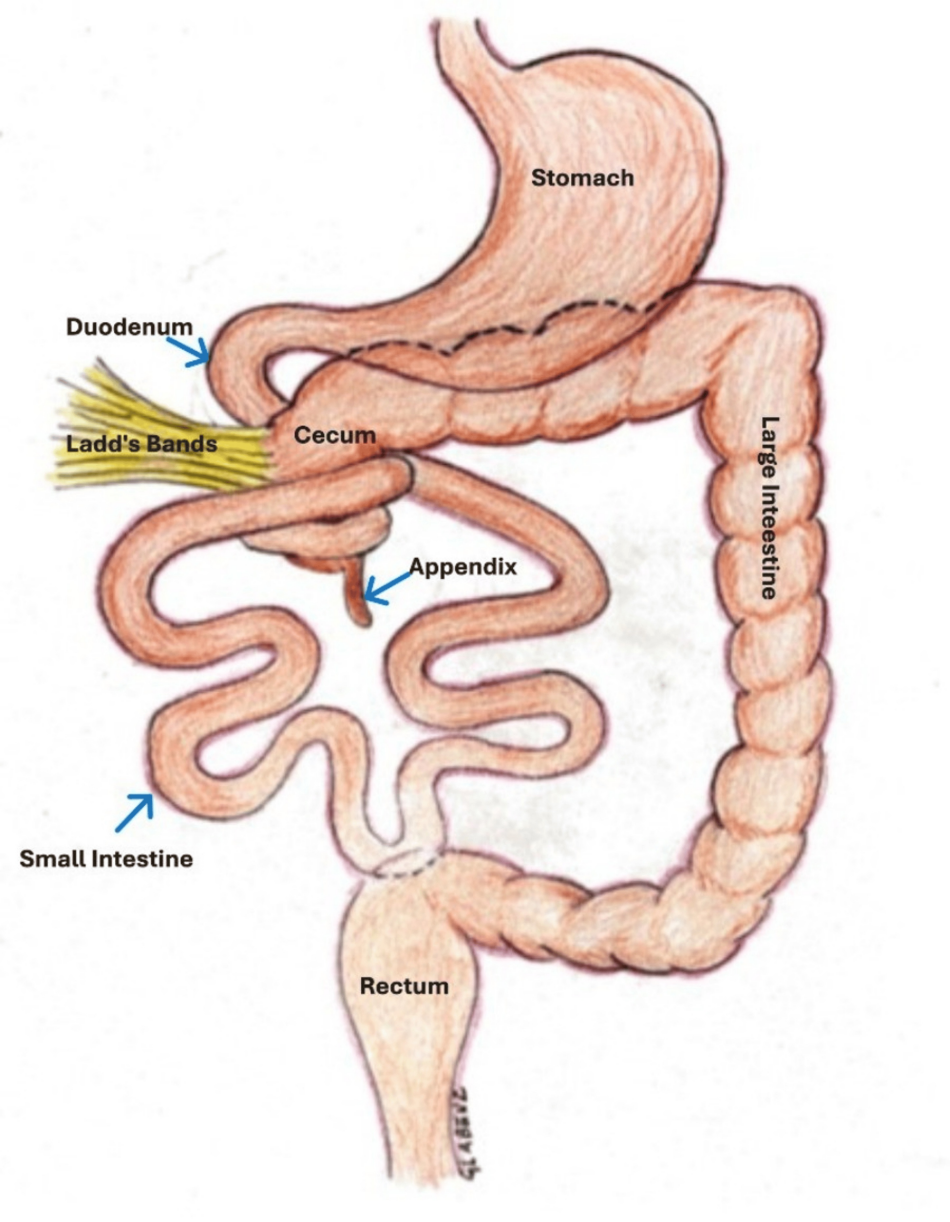
Findings at surgery Schematic representation of operative findings. Drawing is courtesy of Mr. Gregory Labenz, April 2025.

**Figure 5:**
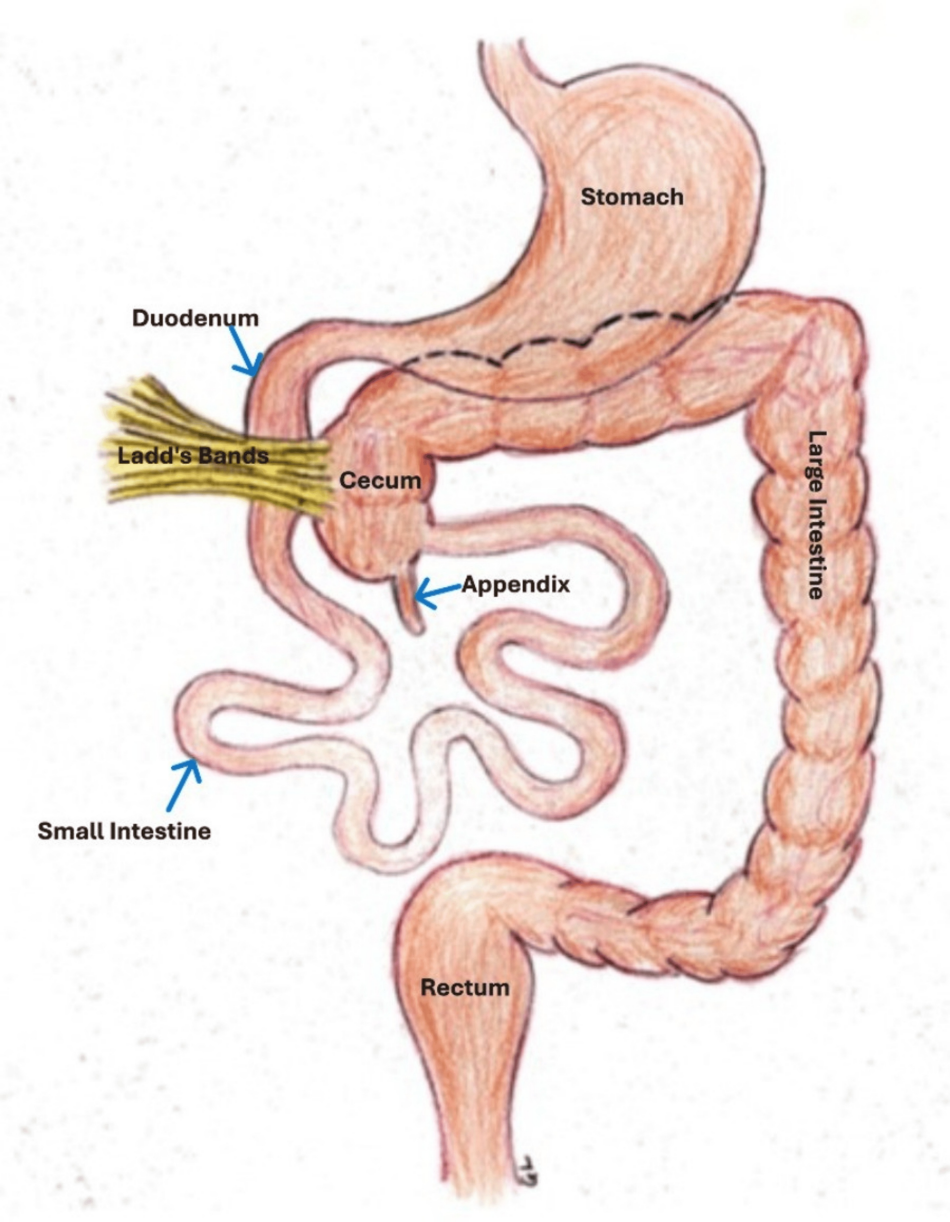
Detorsion Schematic representation of procedure details. Drawing is courtesy of Mr. Gregory Labenz, April 2025.

**Figure 6:**
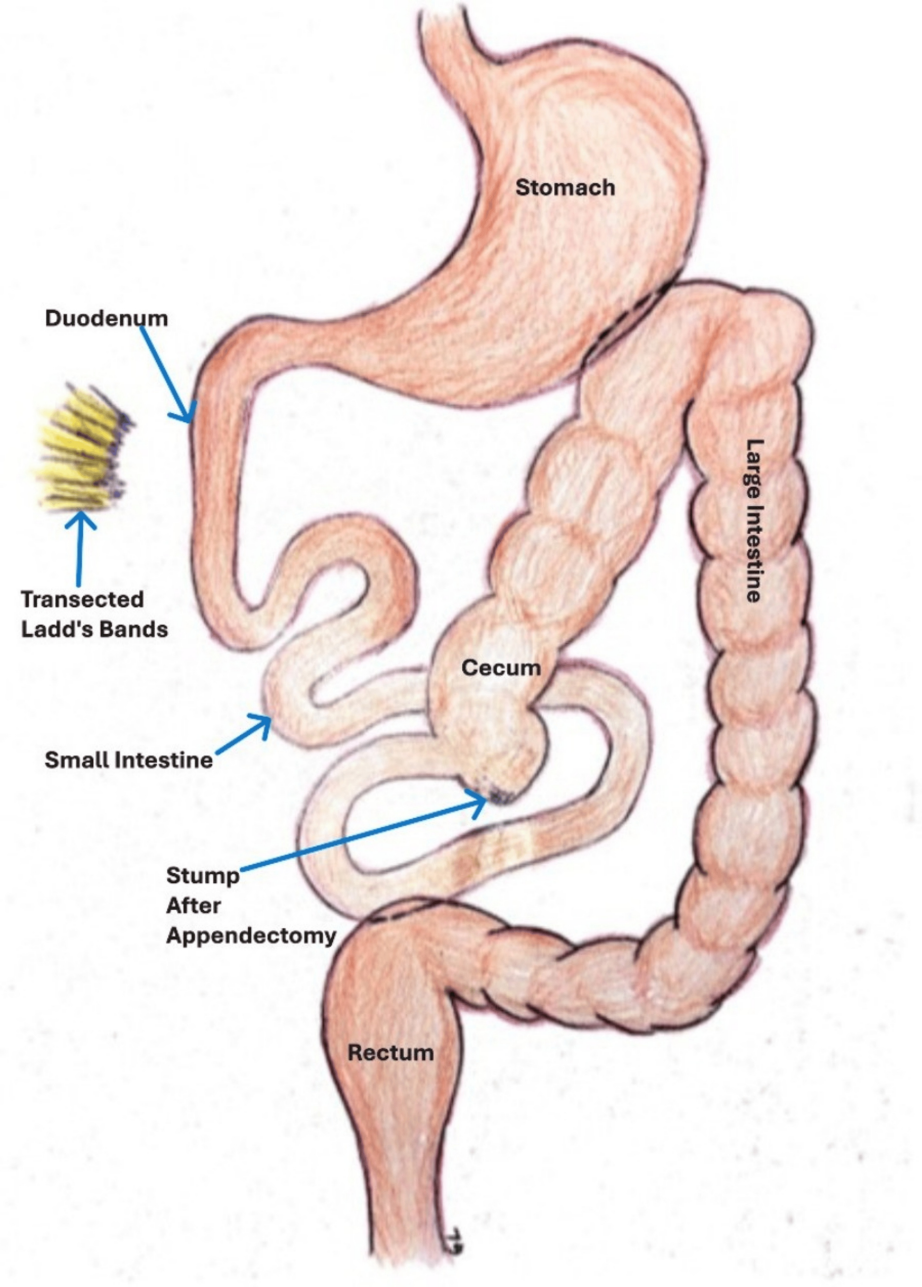
Final operative results Release of bands and broadening of the mesentery, along with appendectomy. Drawing is courtesy of Mr. Gregory Labenz, April 2025.

Over the next 24 hours, the patient was resuscitated with intravenous fluids, and his abdominal distension improved with nasogastric tube decompression. We performed a diagnostic laparoscopy to assess the small bowel obstruction. During laparoscopic surgery, malrotation was suspected, and an open laparotomy was performed. Upon further exploration, Ladd’s bands were discovered with torsion (Figure 4). There was no evidence of volvulus, ischemia, or perforation. The small bowel was detorsed (Figure 5). The Ladd’s bands were lysed, and the small bowel was then placed on the right side of the abdomen, and the colon was placed on the left side with the cecum in the left upper quadrant, which allowed for broadening of the mesentery (Figure 2, Figure 3, and Figure 6). An appendectomy was also performed following a classic Ladd’s procedure [5,6]. This is done to prevent difficulty in diagnosing appendicitis, should it occur, since the appendix is now on the left side.

The patient tolerated the operation well. He had an extended postoperative inpatient admission for management of his renal failure and new-onset atrial fibrillation with rapid ventricular response. Given persistent distension and slow return of bowel function, repeat cross-sectional imaging of the abdomen was performed on the fifth postoperative day. The findings were consistent with a postoperative ileus with no transition point. The CT scan from postoperative day five clearly showed a correction of the "mesenteric swirl" and new position of the cecum in the left upper quadrant (Figure 2b and Figure 3b). The patient regained his bowel function and was discharged on the tenth postoperative day. Upon discharge, the patient was seen by his primary care provider and surgeon multiple times over a year and a half and reported no reoccurrence of symptoms.

## Discussion

The gastrointestinal tract rapidly expands between embryonic development's fourth and eighth weeks. During this period, the primitive intestinal loop exits the abdominal cavity and undergoes a 90-degree counterclockwise rotation around the axis of the superior mesenteric artery [7]. As development progresses, the intestine grows outside the abdomen, eventually returning to the abdomen between the eighth and tenth weeks of gestation. Upon relocation, there is an additional 180° counterclockwise rotation, resulting in a total rotation of 270° counterclockwise. Finally, the bowel is securely fixed to the posterior abdomen, with the duodenum anchored by the ligament of Treitz on the left and the cecum fixated in the right lower quadrant [8].

Failure of the midgut to rotate 270° around the superior mesenteric vessels during embryological development results in either nonrotation or malrotation [9]. In cases of nonrotation, the primitive bowel re-enters the abdomen without further rotation, resulting in the small intestine positioned on the right side and the large intestine on the left. Conversely, malrotation involves the cecum relocating to the mid-upper abdomen, anchoring it to the right lateral abdominal wall by peritoneal bands. This displacement occurs due to partial rotation of the cecocolic limb while the duodenojejunal limb remains in a non-rotated position. Peritoneal bands, known as Ladd’s bands, traverse the duodenum and may lead to compression and obstruction of this segment. Such bands were first described by the pediatric surgeon, William Ladd, in the 1930s [5,6]. Complications of malrotation or non-rotation include intestinal obstruction due to the Ladd’s bands, midgut volvulus, and internal hernias.

The most common form of malrotation, typically called “non-rotation,” involves the incomplete rotation of the duodenum and colon, which involves the initial 90° counterclockwise rotation around the superior mesenteric artery, giving rise to small bowel suspension on a narrow mesentery that lacks fixation in the retroperitoneum [5,6]. This malrotation commonly occurs in infants, children, and adults who present later in life. In infants, the primary presenting symptom of malrotation is emesis, whereas in adults, the most reported symptoms are abdominal pain followed by emesis, nausea, and diarrhea [4]. Severe vomiting in infants may lead to consideration of congenital anomalies, but malrotation is not typically suspected in adults presenting with abdominal pain, vomiting, and nausea in contemporary clinical practice. Furthermore, the clinical presentation of malrotation in adults can be heterogeneous or asymptomatic [10-12], and many adults lack acute catastrophic consequences such as volvulus or obstruction [13]. Therefore, there is a low index of suspicion for malrotation in adults, with the diagnosis often made incidentally.

Several imaging features can indicate malrotation. On abdominal X-ray, small intestinal loops predominantly on the right side without cecal gas indicate malrotation [14]. An abdominal X-ray may also show the classic “double bubble” sign, but this is not present in every case of malrotation [2]. A CT scan has a diagnostic accuracy of 80% and has a variety of indicative findings [15]. These include abnormal position of the duodenojejunal junction, underdevelopment/absence of the uncinate process of the pancreas, or abnormalities in the orientation of the superior mesenteric artery in relation to the superior mesenteric vein. The “whirlpool sign” is also seen during acute volvulus [12]. An upper gastrointestinal series (UGIS) is reported to have a diagnostic accuracy of 80% or greater [13]. UGIS primarily shows abnormal positioning of the duodenojejunal junction, right colon, and cecum [12].

The diagnosis of malrotation in our patient was confirmed by CT scanning of the abdomen and pelvis. Findings included the small intestine on the right side of the abdomen, the large bowel on the left, and the cecum in the upper right quadrant, typical of the 90° counterclockwise rotation of the embryonic bowel without further rotation. When these findings were initially observed, the radiologist and I interpreted them as consistent with a previous right hemicolectomy with a probable stricture of the ileocolonic anastomosis. The possibility of a malrotation variant was not initially entertained. After all, our patient was 80-plus years old; why would such an elderly person have a malrotation problem? However, he denied any previous abdominal surgery and had no scars on his abdomen to suggest such a possibility. It was only after exploration to deal with his bowel obstruction that malrotation was confirmed. This sequence of events conveys the important message that sequelae from congenital malrotation can occur at any age and should always be part of one’s thinking when diagnosing problems involving the abdomen.

Precisely why some patients can possess a congenital malrotation disorder of the gut from birth and not exhibit any clinical symptoms for many decades, even into the ninth decade of life, like our patient, remains unknown. Further, the clinical presentation and factors influencing it, such as volvulus versus bowel obstruction, are also uncertain. Midgut volvulus primarily occurs secondary to a narrow mesentery base prone to clockwise twisting. It is likely that the lifetime risk of volvulus is directly related to the width of the attached mesentery, and that the degree of attached mesentery is likely related to how much rotation was completed prior to embryologic arrest. Those with a very narrow base of mesentery will likely present in infancy with midgut volvulus, and those with a larger base of mesentery may present in older age, if at all. Additionally, patients with a wider base of mesentery attachment may present with chronic midgut volvulus rather than an acute catastrophic episode. This is due to intermittent or only partial twisting of the bowel and may be responsible for the chronic symptoms of malrotation in older patients [16]. However, while unlikely, the lifetime risk of acute volvulus must not be discounted in older patients. This is demonstrated by two cases of malrotation with midgut volvulus in an 80-year-old man and an 83-year-old man [17].

Another variable contributing to the age of presentation may be the degree of extrinsic compression of the bowel by Ladd’s bands. Severe compression leads to acute obstruction in infancy, whereas partial compression may lead to chronic obstructive symptoms. The degree of constriction is likely related to the degree of rotation completed prior to embryologic arrest (i.e., position of cecum in the abdomen). Another possibility is the material intrinsic to Ladd’s bands. Fibrous, stiff bands may lead to more compression than soft, flexible bands. This is difficult to determine, as case reports of malrotation have not commented on the physical composition of Ladd’s bands. The Ladd’s bands in our patient were soft overall and not particularly stiff.

Finally, as seen in our patient, malrotation poses a significant risk of internal hernias. These can form when the colon and duodenum mesenteries are not fixed to the retroperitoneum. A paraduodenal hernia can occur if the cecocolic loop rotates and the duodenojejunal loop does not rotate. While volvulus is traditionally seen as the most prominent consequence, internal hernias are a well-documented risk that appears to be more exclusive to delayed presentations of malrotation [1,18]. This is also demonstrated by a case report very similar to our own, documenting an 84-year-old man with a new diagnosis of malrotation also presenting with an acute internal hernia [19]. Therefore, our case appears to align with contemporary literature suggesting that internal hernias are complications more often seen in older patients with malrotation.

## Conclusions

Our case presentation highlights the importance of considering malrotation as a potential diagnosis in adults with nonspecific abdominal symptoms, regardless of age. This should especially be considered in the case of abnormal imaging findings and signs of obstruction without a history of abdominal surgery or inflammatory bowel disease. Diagnostic modalities such as abdominal X-ray, CT scan, and UGIS play crucial roles in identifying malrotation. However, the condition may still be diagnosed for the first time intraoperatively. Surgical intervention in the form of Ladd’s procedure remains a mainstay of treatment for malrotation. Though conflicting opinions exist, many advocate for treating a symptomatic patient to prevent serious complications such as volvulus. Our case underscores the importance of maintaining a high index of suspicion for malrotation across all age groups, advocating for early recognition, appropriate diagnostic evaluation, and timely intervention to optimize patient outcomes.
